# Affective response to physical activity as a deep phenotype in a non-randomized pilot study

**DOI:** 10.1038/s41598-022-09662-3

**Published:** 2022-04-07

**Authors:** Harold H. Lee, John E. McGeary, Shira Dunsiger, Jessica A. Emerson, Beth Bock, Jeanne McCaffery, Kayla Dwyer, Angela D. Bryan, David M. Williams

**Affiliations:** 1 Harvard University TH Chan School of Public Health, Boston, MA USA; 2 University of North Carolina at Chapel Hill, Chapel Hill, NC USA; 3 Providence Veterans Affairs Medical Center, Providence, RI USA; 4 Warren Alpert Medical School of Brown University, Providence, RI USA; 5 Brown University School of Public Health, Providence, RI USA; 6 The Miriam Hospital, Providence, RI USA; 7 University of Connecticut, Storrs, CT USA; 8 University of Colorado Boulder, Boulder, CO USA

**Keywords:** Human behaviour, Risk factors, Genetic predisposition to disease

## Abstract

Large-scale genomic studies are beginning to identify genetic predictors of physical activity (PA). For those genetically predisposed to engage in low PA, a behavioral intervention may target a malleable factor that mediates genetic predisposition to low PA (i.e., intermediate phenotype) to mitigate the genetic influences. In a non-randomized exercise promotion pilot study, we test the feasibility of examining affective response to PA (how one feels during PA) as an intermediate phenotype between genetic variation and PA adherence. We hypothesized that three single nucleotide polymorphisms (SNPs; rs8044769 and rs3751812 in FTO; rs6265 in BDNF), identified from a prior systematic review, would be predictive of affective response to PA, and that affective response to PA would mediate the SNP-PA link. Forty five healthy, low-active adults received a 12-week print-based PA promotion program. Baseline affective response to PA was assessed using the Feeling Scale, a single-item measure of affective valence. Moderate to vigorous PA (MVPA) was assessed using accelerometers pre- and post-intervention. We examined the three SNPs in a weighted genetic score. Age, sex, body mass index, race, and neighborhood walkability were potential covariates. Affective response to PA and MVPA at follow-up (minutes/day over 4–7 days) were regressed on variation in SNPs, controlling for covariates. One unit increase in genetic score was associated with a 0.14 higher mean Feeling Scale, though was not statistically significant (*p* = 0.13). Among individual SNPs, having an additional *FTO* rs8044769 C allele was associated with a mean Feeling Scale score of 0.53 units higher (*p* = 0.015), which was statistically significant after applying the corrected *p*-value of 0.016. The genetic score or individual SNPs were not predictive of MVPA 12 weeks later, thereby mediation analyses were not performed. The preliminary findings demonstrate the promise of the intermediate phenotype approach.

## Introduction

Physical inactivity is the fourth leading risk factor for global mortality, accounting for 9% (5.3 million) of premature deaths globally^1^. However, only half (51.6%) of US adults meet the national guidelines of expending ≥ 1000 kcals/week through physical activity (PA)^2^. Thus, understanding the motivation for PA is imperative for public health.

Genomic information can predict a sizable proportion of variance (50-78%) in PA^3–14^, and recent genome-wide association (GWA) studies of PA are beginning to identify PA-related single nucleotide polymorphisms (SNPs)^15–17^. Given the rapidly advancing large-scale genomic data collection efforts^18,19^, it is reasonable to speculate that researchers will soon accrue substantial knowledge of the SNPs that, together, can predict a nontrivial proportion of variation in PA. However, most (or all) GWAS of PA use relatively shallow phenotypes, such as naturally occurring PA level at a single time point, which yields insufficient knowledge regarding how to change PA behavior. To overcome the weaknesses of the GWAS approach, one can take a deeper phenotyping approach, which refers to collecting more precise^20^, fine-grained^21^, and mechanistically comprehensive^22^ data regarding phenotypes than the shallow phenotypes. In the context of PA phenotypes, a malleable phenotypic factor that mediates genetic influence on PA behavior can be considered a deeper phenotype. Identifying such intermediate phenotypes can be useful because they can be targeted by PA promotion interventions for those who are genetically predisposed to engage in lower PA levels. Moreover, since an intermediate phenotype is a more proximal variable to the target behavior, it is relatively less susceptible to non-genetic factors confounding the target behavior (e.g., build environment’s effect on PA level), constituting a conceptually pristine phenotype likely resulting in high fidelity empirical data. Such intermediate phenotype approaches have been employed in various health behaviors, including subjective and affective responses to the consumption of alcohol^23,24^, caffeine^25–28^, amphetamines^29–31^, and nicotine^32–35^.

For PA promotion, a conceptually plausible intermediate phenotype, which is also supported by a wealth of empirical studies, is affective response to PA (i.e., how one feels during PA). Humans and animals tend to repeat behaviors that feel good and avoid behaviors that feel bad^36–40^. This ancient principle of psychological hedonism (e.g., Bentham’s Utilitarianism^41^) was resurrected in late 20th century by Nobel Laureate, Daniel Kahneman^39^, whose work precipitated the paradigm shift in behavioral scientist’s focus from *reasoning* (e.g., “Exercise is good for our health”) towards *feeling* (e.g., “I do not exercise because it feels bad”). Nested in psychological hedonism, *affective response to* a behavior is defined as the shift in core affective valence, which ranges from extreme negative valence (i.e., displeasure) to extreme positive valence (i.e., pleasure) with a middle neutral point^42^. Positive affective response refers to a positive shift on the affective valence dimension, whereas negative affective response means a negative shift in affective valence, from immediately *prior to* a behavior to *during the* behavior^42^. Consistent with psychological hedonism, those who experience a more positive affective response to PA are more likely to repeat it in the future and thus more likely to engage in regular PA, according to a systematic review of empirical studies^43^. Thus, if affective response to PA is shown to be a viable intermediate phenotype, it can advance understanding of the genetic basis of PA behavior and potentially provide new opportunities for PA promotion interventions.

Thus, the purpose of this study was to test the feasibility of examining affective response to PA (i.e., how one feels during PA) as a potential intermediate phenotype. We examined three alleles that are associated with affective response to PA from a prior systemic review^44^: A allele of rs6265 in the Brain-Derived Neurotrophic Factor gene (*BDNF)*, and T allele of rs3751812 and C allele of rs8044769 in Fat Mass and Obesity-related gene (*FTO*). Notably, C allele of rs8044769 *FTO* is associated with higher obesity; this is counter intuitive because, if the C allele is associated with a more positive affective response to PA it should lead to a higher volume of PA, ultimately functioning towards preventing weight gain. Despite the limited biological plausibility, we did include the rs8044769 in our analysis because, *a priori*, we planned to examine SNPs selected from the systematic review^45^, which was conducted because there are no GWAS of affective response to PA conducted. Although the present pilot study was not fully powered, we had *a priori* preliminary hypotheses to see whether we can detect small signals from the SNPs. We hypothesized that we would replicate prior findings, with each selected allele associated with a more positively valenced affective response to PA (i.e., feeling ‘good’ versus ‘bad’), and that affective response to PA will mediate the association between the SNP(s) and future PA behavior. We controlled for known sociodemographic factors related to PA, including age, sex, body mass index, and race.

## Results

### Descriptive statistics

**Figure 1:**
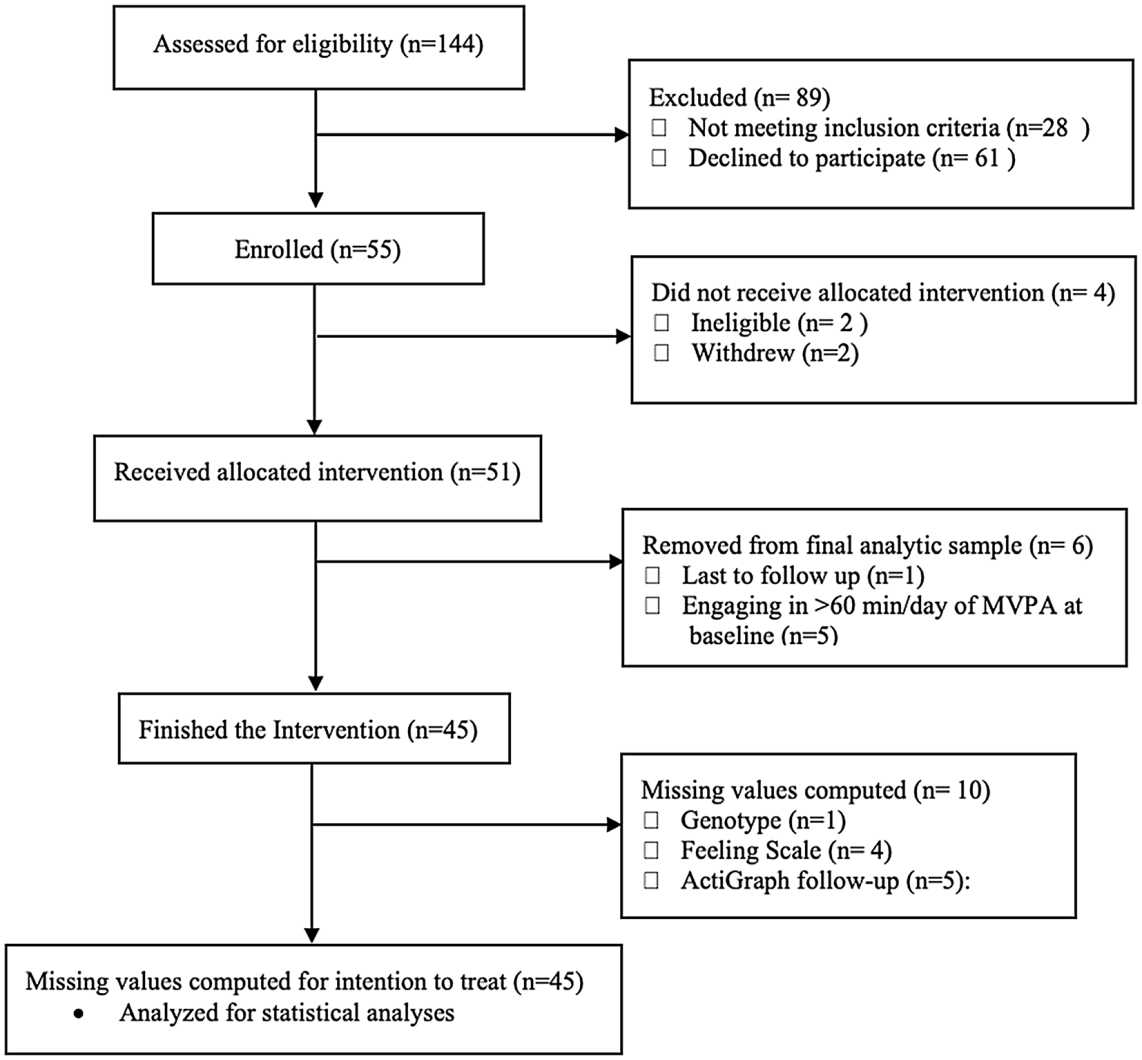
Flow Diagram.

A total of 144 adults were assessed for eligibility, and 55 signed consent forms. Of those, 51 received the 12-week long intervention, and 45 finished the final follow-up assessment at week 12 (Fig. 1). The data from these 45 participants constituted the final sample for analysis (Table 1). Participants were mostly female (71%), white (78%), had college or post-graduate education (77%), were not married (69%), and were without children under 18 living with them (82%). Nearly half of the sample earned more than $40,000/year (42%), and over a quarter were unemployed (22%). On average, participants were about 34 years old (*M*=34.1, *SD*=11.5), with body mass index (BMI) in the overweight category (*M*=28.1, *SD*=6.1). The mean daily moderate to vigorous PA (MVPA) difference from pre (22.2 ± 14.3 minutes per day) to post-intervention (22.7 ± 15.8 minutes per day) was not significant (*t*=-0.19, *p*=0.84, *df*=44), suggesting that the intervention did not increase the mean daily MVPA level. However, there was between-subjects variability in changes from pre- to post-intervention, with 28% increasing MVPA more than 5 minutes per day, 36 % decreasing more than 5 minutes per day, and 36% remaining the same (i.e., daily MVPA change was less than 5 minutes). While the affect during PA was normally distributed with a median value of 1.3 (Supplementary Fig. 1), affective response to PA (i.e., change in affective response from before to during moderate-intensity PA) was normally distributed with a median value of 0 (Supplementary Fig. 2). Put differently, participants on average reported that they felt positive during moderate-intensity PA. However, when this value was compared with affect before exercise, roughly half of participants’ affect *shifted* negatively (e.g., Feeling scale score shifting from 3 to 2, 1 to 1, or 1 to −1); it is this variability in affective response to exercise that has been shown to be predictive of future exercise behavior and thus was the focus in the present study. In models only adjusting for baseline affect, average affect during moderate intensity exercise was associated with BMI (β=0.05, *p*=04), obesity (β=0.78, *p*=0.009**)**, age (β=0.02, *p*=0.04) but not sex (β_female_=-0.15, p=0.94). Genotype distribution was satisfied in the Hardy Weinberg Equilibrium assumption (rs6265: *χ*^2^ = 0.038, *p*=0.84; rs3751812: *χ*^2^ = 0.059, *p*=0.44; rs8044769: *χ*^2^ = 1.27, *p*=0.26).

**Table 1:** Population Characteristics (n = 50).

Characteristic	Total (n = 45)
Age (year)	34.1 ± 11.5
Women	32 (71)
Body mass index (kg⋅m^-2^)	28.1 ± 6.1
White	35 (78)
Hispanic	5 (11)
**Education**	
High school graduate	2 (4)
Some college	7 (20)
College graduate	22 (46)
Post-graduate work	14 (31)
**Income**	
Under $10,000	3 (7)
Between $10,000–19,999	3 (7)
Between $20,000–29,999	3 (7)
Between $30,000–39,999	9 (20)
Between $40,000–49,000	7 (16)
Over $50,000	16 (36)
Don’t know or Prefer not to answer	2 (4)
Marital Status	36 (58)
Single	14 (31)
Married/Partnered	5 (11)
Separated/Divorced	36 (58)
Employed	35 (78)
**rs6265 (%)**	
GG	35 (78)
GA	9 (20)
AA	1 (2)
**rs3751812 (%)**	
GG	21 (47)
GT	17 (38)
TT	7 (16)
**rs8044769 (%)**	
TT	14 (31)
TC	18 (40)
CC	13 (29)

### Genetic variation and affective response to PA

**Figure 2:**
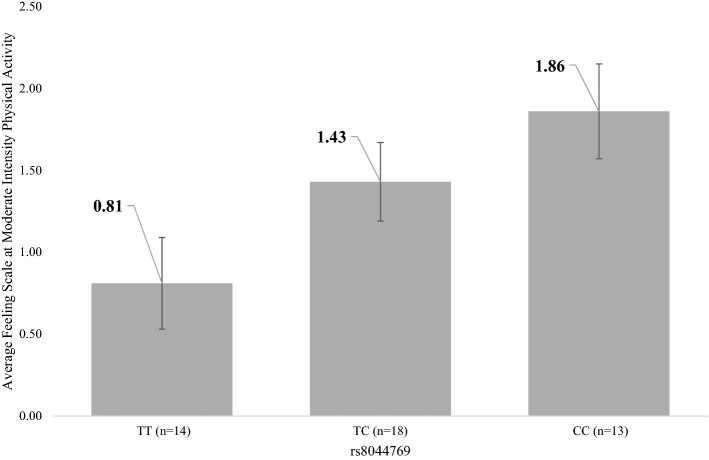
*FTO* SNP rs8044769 and Average Feeling Score at Moderate Intensity Physical Activity based on adjusted regression model in Table 1. The predicted mean value from a multivariate regression model, in which average Feeling Scale report during moderate intensity physical activity was regressed on *FTO* SNP, rs8044769, adjusting for Feeling Scale report before physical activity, age, sex, race, and body mass index.

One unit increase in genetic score was associated with 0.14 higher mean Feeling Scale, though was not statistically significant (*p *= 0.13). One SNP in the *FTO*, rs8044769, was associated with affective response to PA. In the model only adjusting for baseline affect, having an additional *FTO* rs8044769 C allele was associated with a mean Feeling Scale score of 0.48 units higher compared to participants with TT genotype of rs8044769 (*p *= 0.019). After adjusting for all covariates, having an additional C allele was associated with a mean Feeling Scale score of 0.53 units higher (*p *= 0.015) (Fig. 2), which was statistically significant after applying the corrected p-value of 0.016. To address population stratification, we performed the same analyses among Whites only (n = 35). The result showed a stronger effect, such that having additional C allele of rs8044769 was associated with a mean Feeling Scale score 0.75 (vs. 0.53 in the primary analysis) units higher (*p *= 0.006). The rest of the two SNPs, rs6265 and rs3751812, were not associated with affective response to exercise.

### Mediation model and direct effect (genetic variation and future physical activity)

The genetic score was not predictive of MVPA perday at follow-up (*p *= 0.67). Contrary to expectations and prior literature, affective response to moderate intensity PA at baseline was not predictive of average MVPA per day at follow-up (*p *= 0.95). In post-hoc analyses, we examined whether affective response to PA is associated with 5min increase in daily MVPA vs. others (i.e., those that remained the same or decreased), and the affective response to PA was not associated with the binary MVPA outcome in the full model (*p *= 0.12) and model only adjusting for baseline affect (*p *= 0.27). Thus, we did not proceed further with mediation analyses.

### Post-hoc analyses

Since *FTO* is an adiposity gene, BMI may influence the results. Thus, we performed *post-hoc* analyses to examine whether BMI varied by *FTO* SNP, rs8044769. We also examined whether MVPA varied by BMI. The rs8044769 SNP was not associated with baseline BMI (*p *= 0.33) or MVPA at follow up (*p *= 0.71).

## Discussion

Our objective was to test the feasibility of examining affective response to PA (how one feels during PA) as an intermediate phenotype between genetic variation and PA adherence in a non-randomized exercise promotion pilot study. As hypothesized, one SNP in the *FTO* gene, rs8044769, and the genetic score were associated with affective response to PA. However, there was no significant direct effect of the candidate gene factors on PA behavior, nor was affective response to PA predictive of PA behavior.

Our findings replicate a prior candidate gene study by Karoly et al.^46^, who examined the association between the same *FTO* SNPs and affective responses to PA among inactive but otherwise healthy adults. Participants performed a 30-minute submaximal aerobic exercise session (i.e., 65% of participant’s previously established VO_2 max_), with affect assessed immediately before the session and 10, 20, and 30 minutes into the session. They found that the same alleles of the *FTO* SNP (C of rs8044769) were associated with a positive shift in affective valence during PA. However, in a prior GWA study, the C allele of rs8044769 *FTO* SNP—which is related to a positive affective response to PA, and hence, should lead to a higher volume of PA in the future—was, counterintuitively, reported to be associated with higher BMI^47^; similarly, another obesity prone *FTO* SNP (i.e., A allele of rs9939609) was associated with being more physically active compared to non-carriers in 71 Scottish children 4 to 10 years of age.

*FTO* SNPs has been extensively examined with PA, although most of the studies examined obesity as the major outcome with PA as a moderator (i.e., *FTO* × PA BMI)^48^. In contrast, we examined PA (vs. BMI) as the major outcome and psychological phenotype as a mediator. Moreover, we only found associations with psychological phenotype. In this light, more insights may be gained from comparing our findings with a more recent body of *FTO* studies that examined psychological phenotypes, such as depression. In fact, a recent review on the *FTO* gene with comorbidity of depression and obesity points to the imbalanced research of *FTO* (i.e., a much larger volume of research on BMI than depression), calling for more research on *FTO* and depression. For example, prior research has shown that the obesity-prone allele of *FTO* SNP (e.g., A allele of rs9939609) was associated with a higher likelihood of becoming depressive, independent of BMI^49^, likely reflecting a shared genetic influence of *FTO* gene on phenotypes (i.e., pleiotropy on depression and BMI). Taken together, given that the obesity-prone *FTO* alleles were associated with a more positive affective response to PA in the present study (controlling for BMI), it is plausible that the carriers of the obesity-prone *FTO* alleles are generally depressed. Thereby, participation in moderate-intensity PA may be functioning as an opportunity to come out of the depressiveness, which manifested as a positive affective response to exercise in our study. However, this is speculation based on prior literature. Besides, given effects of genetic variations on psychological, behavioral, and anthropometric phenotypes are polygenic (i.e., multitudes of SNPs contributing small effects), adequately powered genetic studies with a much greater number of SNPs (e.g., the polygenic scores of PA, depression, and BMI) will yield stronger insights regarding how to maximize usage of genotype information for changing behaviors.

Unexpectedly, affective response to PA at baseline was not predictive of MVPA at follow-up. This finding is contrary to five previous longitudinal studies that have shown that affective response to PA is predictive of future PA^50–54^. It is possible that this discrepancy can be explained by an affect-age interaction, such that the effect of affective response to PA on future PA may be more salient among the middle-aged adults and adolescents examined in prior studies (19–21), but not among the younger adults who participated in the present study.

The present study has limitations. The sample size was small, quite severely compare to GWAS studies. Moreover, more PA-related SNPs are identified (4–6), which were not included in the present study as they were identified after the genotyping was conducted. Finally, a strong discrepancy in MVPA level at screening (assessed by self-report) and baseline (assessed by accelerometer) was observed. Future studies examining MVPA level should pay close attention to assessment reactivity as well as harmonizing assessment tools for screening and the study. The strengths of the present study are that we chose SNPs from a systematic review and objectively assessed MVPA using accelerometry. Additionally, affect was assessed using a smartphone app to minimize social desirability.

In theory, an intermediate phenotype (e.g., affective response to PA) should constitute a high-fidelity phenotype with less noise as it is less susceptible to non-genetic factors (e.g., built and social environment) that confound the behavioral phenotype (i.e., PA). We empirically demonstrated the promise of the intermediate phenotype approach, given that the major finding (i.e., C allele of *FTO* SNP, rs8044769, was associated with affective response to PA) supports our *a priori* preliminary hypothesis and replicates a prior candidate gene study^46^. However, it is critical to note that the major finding *per se* should be interpreted with caution due to the extremely low sample size. Moreover, the conflicting findings (i.e., obesity prone allele’s association with positive affective response to PA) may reflect a shared genetic influence on phenotypes (i.e., pleiotropy on depression and BMI), which warrant future investigation in studies with sufficiently large sample sizes with greater number of SNPs (e.g., PA-related SNPs (4–6); polygenic risk score of BMI and depression).

## Methods

### Participants

All methods were carried out in accordance with the CONSORT 2010 Statement. Final analytic sample included 45 men and women ages 18-65 who were healthy, underactive (MVPA min/week < 60) based on telephone screening by research staffs using a self-reported questionnaire^55,56^, finished the intervention (Fig. 1). They were recruited from the greater Providence, RI area through print (newspaper, flyer) and internet (Facebook, CraigsList) advertisements. Exclusion criteria included various physical conditions that may make exercise unsafe, such as medical conditions of the heart (e.g., cardiovascular disease, hypertension), lungs (e.g., COPD, uncontrolled asthma, emphysema), daily psychoactive drug use (e.g., marijuana) or presence any other substance use disorder, and any other physical limitation that would make exercise unsafe (e.g., orthopedic problems, osteoarthritis). All participants read and signed an informed consent form that was approved by the institutional review board at Brown University (#1604001464). All experiments were performed in accordance with relevant guidelines and regulations. Participants were enrolled between December 2017 and September 2018, with final follow-ups conducted in December 2018 (ClinicalTrials.gov Identifier: NCT03415542, 30/01/2018).

### Design and procedures

The present study used a longitudinal observational design. The PA promotion intervention was provided to all participants without randomization to create change in PA because, without such intervention, individuals' PA levels were unlikely to change over time. Accordingly, all participants were enrolled in a 12-week print-based PA promotion program.

We collected a saliva sample, assessed affective response to moderate intensity PA through treadmill walking, and provided instruction for the accelerometry in the initial visit. At the end of this visit, we provided ActiGraph accelerometers (wGT3X-BT) to assess baseline PA. Participants were asked to wear the device for at least 10 hours a day for 7 days. Since we attempted to measure baseline PA before the intervention, research staff explicitly told participants to maintain their usual exercise level (i.e., inactive or less than 60 minutes per week of moderate-intensity exercise based on participants’ self-report during screening). Nonetheless, according to the accelerometer data at baseline, the vast majority of participants (84%) were engaging in more than 8.57 minutes per day of MVPA (60 min/7days = 8.57 min). In other words, most of the participants (>80%) were meeting the guideline for MVPA at baseline. It is possible that, just by enrolling in the study, some participants may have enhanced their motivation to engage in MVPA (i.e., assessment reactivity) during the baseline period, in which MVPA was measured using accelerometers. To reduce the impact of outliers (i.e., extremely physically active participants), we excluded 5 participants who were engaging in more than 60 minutes per week of MVPA from the analytic sample.

 Participants were asked to download the free MetricWire app (www.metricwire.com), free to download on iOS and Android smartphones. Using the app downloaded on the participant's smartphone, participants reported their affect before and during treadmill walking. These variables were further operationalized as the intermediate phenotype of the present study, affective response to PA (detailed in *Measures* section). After wearing the ActiGraph for one week, participants came back with their ActiGraph. In this visit, all participants received the PA promotion intervention. Brief exercise counseling about safe exercise practices, along with a printed pamphlet, were provided to participants prior to the intervention, including (a) ways to monitor their exercise intensity using the Rating of Perceived Exertion Scale^57^ and (b) recommendations to increase exercise intensity over time gradually.

All participants received a standard version of a print-based PA promotion program. Participants received a printed document in the mail at weeks 1, 5, and 11, which was designed to help them overcome barriers to PA. Phone interviews were conducted at week 5 and week 11. Interventionists (JE and HL) both hold master's degrees in behavioral and social sciences and received training in motivational interviewing through a workshop. To minimize provider difference, all week 5 interviews were conducted by JE, and all week 11 interviews were conducted by HL. The phone interview lasted about 10-15 minutes. At the end of week 12, participants attended the final in-person session to return the ActiGraph after wearing it for 7 days. Participants were asked to wear the device for at least 10 hours a day.

### Measures

Baseline assessments included a saliva sample for DNA analysis, questionnaires assessing sociodemographic information and neighborhood walkability, and a 30-min moderate intensity (i.e., 64–76% of maximum heart rate [i.e., 220–age]) treadmill walk to assess affective response to PA. Affect was assessed before and during (every 4 minutes for 30 minutes) the treadmill walk via a smartphone app in order to reduce social desirability bias that may have occurred as a result of reporting affective response directly to research staff. Free-living PA behavior was assessed over 7-day intervals via accelerometry at baseline and at the end of the intervention.

We performed genotyping for three SNPs (rs6265, rs8044769, and rs3751812). Saliva samples were stored in a −70 °C freezer in a locked laboratory, initially at the Center for Alcohol and Addiction Studies at Brown University and then transferred to the Providence Veterans Affair Medical Center for analysis in Dr. McGeary’s lab. Genomic DNA was extracted from saliva samples and assayed by real-time PCR using TaqMan SNPs genotyping assay (ThermoFisher Scientific Assay ID: C_11592758_10 for rs6265, C_29387698_10 for rs8044769, and C_27476887_10 for rs3751812).

Detecting genetic effects on phenotype can be masked if genetic effects are manifested only in predisposing environments. Thus, the environmental influence of PA was assessed by the 17-item Neighborhood Environment Walkability Scale (16). The test-retest reliability of this measure ranges from 0.52-0.81, and a validity test with a similar, but longer survey, Neighborhood Environment Walkability Scale, ranges from 0.27-0.81 (16).

Affective response to PA was measured before and during the baseline treadmill protocol using the Feeling Scale (14), a single-item measure of core affective valence ("How are you feeling right now?") with response options of −5 = very bad, −3 = bad, −1 = somewhat bad, 0 = neutral, +1 = somewhat good, +3=good, and +5=very good. From the multiple measures, we computed the mean value of Feeling Scores when heart rate is 67–73%, which was operationalized as affective response to PA.

Accelerometers (ActiGraph wGT3X-BT monitor) were used to measure average MVPA per day, which was computed by summing the number of minutes in MVPA dividing it by the number of valid days (i.e., “Average.MVPA.Per.Day” produced from the ActiLife software). ActiGraphs were worn on each participant's hip over 4-7 day periods at baseline and at the end of the intervention, for at least 10 hours per day, consistent with standard practice in the field (15).

### Statistical analyses

Hardy Weinberg Equilibrium was determined using *χ*^2^. In individual SNP analyses, genotypes were grouped as additive, dominant, and recessive models. Cumulative effects of SNPs were examined using a genetic score, which is computed by adding the number of effect alleles from the 3 SNPs (using an additive model) with weights equal to the published per-allele effects. With regard to sample size calculation, 45–74 participants would yield 80% power to detect a relationship between the SNPs with alpha=.05 (with minor allele frequency being .19–.21 among Hispanic and non-Hispanic Caucasian), assuming effect size of *r*^2^=.10 ~.16 based on prior studies.^51,52,58^ Given that genome-wide association studies with a large sample size (>100,000) would identify ~100 SNPs that altogether explain ~3% of phenotypic variation,^59^ the effect size of 10–16% is likely inflated (e.g., Beavis effect or “Winner’s curse”^60^). Notably, compared to typical GWAS phenotypes, intermediate phenotypes are relatively less susceptible to non-genetic factors confounding the target behavior (e.g., built environment), constituting pristine data with reduced mean square error, which upticks statistical power.^61^ Still, the sample size of 50 is likely not powered for a formal hypothesis test, though we had *a priori* preliminary hypotheses to see whether small signals from the SNPs could be detected. For the association between SNPs and affective response to PA, we used linear regression models in which affective response to PA (mean value of Feeling Scores when heart rate is 67–73%) was regressed on each of the three SNPs separately and the covariates, including pre-PA affect (measured 1 minute before treadmill walking), age, sex, race, BMI and the environmental influence on PA (i.e., neighborhood walkability). PA was operationalized as average moderate to vigorous PA (MVPA) per day at week 12, controlling for baseline average MVPA per day. For the association between SNPs and MVPA, linear regression models were used in which average MVPA per day were regressed on the same independent predictors as described previously. Models additionally controlled for accelerometer wear time at follow-up and baseline MVPA. All analyses were run on the intent to treat sample, and missing values were imputed using the predictive mean value using R package, MICE. For individual SNP analyses, a corrected significance threshold of 0.016 (=0.05/3 tests) was used.

## Data availability

All data generated or analyzed during this study are included in this published article (and its Supplementary Information files).

## Supplementary Information

Supplementary Information 1.

Supplementary Information 2.

Supplementary Information 3.
