# Supplementary material for: Affective response to physical activity as a deep phenotype in a non-randomized pilot study
Source: Sci Rep. 2022 Apr 7;12:5893. doi: 10.1038/s41598-022-09662-3 (PMC8989978; doi:10.1038/s41598-022-09662-3)
Supplement: Supplementary file 3 — Supplementary Information 3. [file 41598_2022_9662_MOESM3_ESM.docx]

| 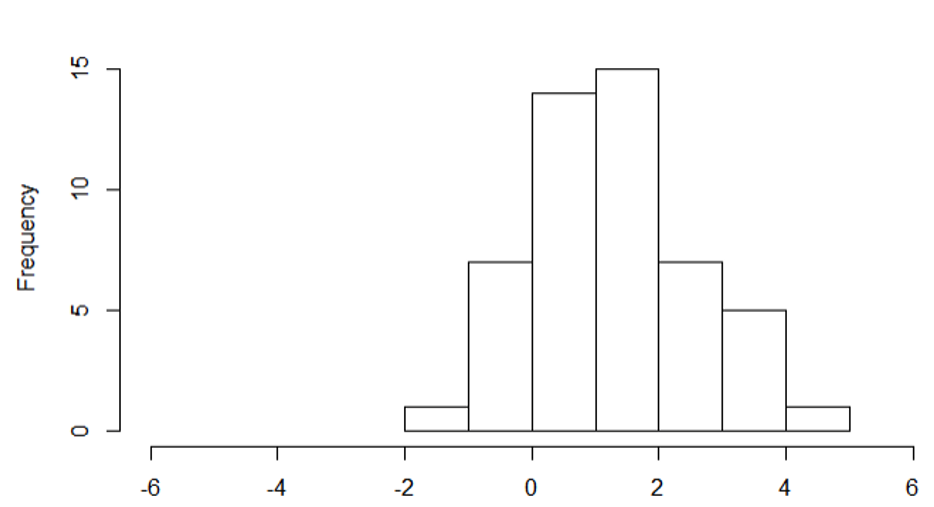 |
| --- |
| **Supplement Figure 1**. Distribution of Affect during Moderate Intensity Physical Activity (=*average Feeling Scale score at moderate intensity physical activity*) Range: -1.5 ~ 4.3, Median = 1.33, Mean = 1.34 |

| 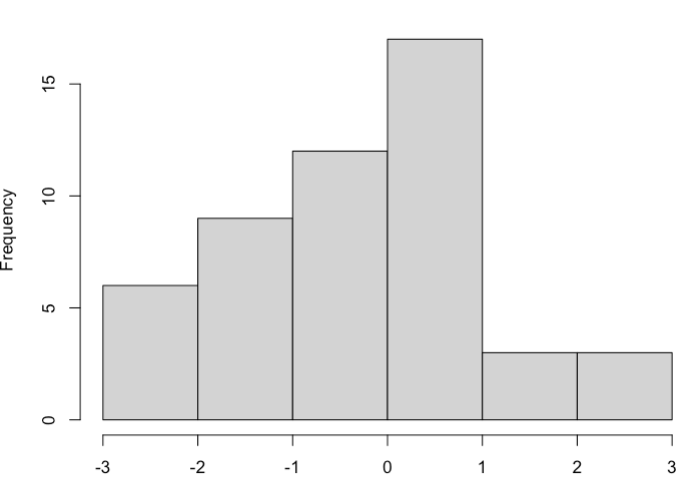 |
| --- |
| **Supplement Figure 2**. Distribution of Affective Response to Physical Activity (= computed by subtracting *average Feeling Scale score at moderate intensity physical activity* by *baseline Feeling Scale score at baseline*). Range: -2.5 ~ 3.0, Median = 0.0, Mean = -0.09 |
